# 

**Published:** 1855-11

**Authors:** 


### NOTICES OF BOOKS, PERIODICALS, &C.

The Scalpel for October is sold, 50 pp. 8vo, of practical truth, for 25 cents. There is too much sound, searching philosophy in the Scalpel to make it pay a dividend to its industrious Editor directly. People don't patronize publications largely, which impart to them substantial and profitable information. Fond papas and weak mothers, which by the way are ninety-nine per cent. of the whole lump, have not the intelligence, which busies itself in rearing healthful sons and daughters; the father looks to a pecuniary match, while the mother is monomanical on the subject of "*set*;" her idea of mundane success and felicity, is to have her child marry upwards, into a "*set*" above her own. Both, father and mother, would heartily agree, for once at least, in the marriage of their child to a leper, moral and physical, provided a higher "*set*" and a fortune were thrown in. "Who shall I marry?" said a clever widower to us yesterday, "I have been looking out for three years." Marry the first healthy, tidy, industrious woman you come across; don't marry a poor, pale, sickly creature, if she is worth a million, and high as the sky as to position.

The 27th Article is well worthy of universal perusal,—"*The Cultivation of Domestic and Social Habits as a Means of preventing Disease.*"

---

The Specialist, No. 2, for October, is received; a medical monthly of 16 p. 4to, double columns, and of inviting mechanical execution. Published by Sherman & Co., No. 1 Vesey st., New York, for one dollar a year. It is "A Journal for Diseases of the Chest." We have not seen No. 1 yet, Oct. 25th, and therefore do not speak of the nature and character of the work, until we have seen what will be the spirit and tone of subsequent numbers. It requires some months for a magazine editor to get into harness.

---

Childs & Peterson, 124 Arch st., Philadelphia, have in press *The Year Book of Agriculture*, being an Annual of Agricultural progress and discovery for 1855, forwarded, pre-paid, per mail, for $1 50, containing as it will a great number of engravings, statistical information on all subjects directly pertaining to Agriculture. Every intelligent farmer should possess himself of a copy.

☞ We will furnish one copy and the *Journal of Health* for one year for Two Dollars; postage fifteen cents.

Bodenhamer on Piles, Fistulas, &c., published by J. S. Redfield, 34 Beekman st., New York; 268 pp. 8vo, with fine engravings, $2. A scientific book, by an educated physician, who writes from the personal observation and experience of twenty-five years on a single class of diseases. In skill and success, Dr. B. has no superior living. In saying this we say much, but no more than we believe to be due. The object of the book is threefold:

To detail the symptoms of the diseases;

To give instruction as to their prevention;

To give information where they may be treated.

We advise those who suffer with these ailments to purchase the book, and then decide for themselves, whether they will apply to the author or not.

☞ This book and the Journal for one year will be furnished for Two Dollars; postage 25 cents.

